# Structural disorder and the loss of RNA homeostasis in aging and neurodegenerative disease

**DOI:** 10.3389/fgene.2013.00149

**Published:** 2013-08-14

**Authors:** Douglas A. Gray, John Woulfe

**Affiliations:** ^1^Centre for Cancer Therapeutics, Ottawa Hospital Research InstituteOttawa, ON, Canada; ^2^Department of Biochemistry, Microbiology, and Immunology, University of OttawaOttawa, ON, Canada; ^3^Department of Pathology and Laboratory Medicine, University of OttawaOttawa, ON, Canada

**Keywords:** intrinsic disorder, RNA-binding proteins, proteasome, aggregation, neurodegeneration

## Abstract

Whereas many cases of neurodegenerative disease feature the abnormal accumulation of protein, an abundance of recent literature highlights loss of RNA homeostasis as a ubiquitous and central feature of pathological states. In some diseases expanded repeats have been identified in non-coding regions of disease-associated transcripts, calling into question the relevance of protein in the disease mechanism. We review the literature in support of a hypothesis that intrinsically disordered proteins (proteins that lack a stable three dimensional conformation) are particularly sensitive to an age-related decline in maintenance of protein homeostasis. The potential consequences for structurally disordered RNA-binding proteins are explored, including their aggregation into complexes that could be transmitted through a prion-like mechanism. We propose that the spread of ribonucleoprotein complexes through the nervous system could propagate a neuronal error catastrophe at the level of RNA metabolism.

## INTRODUCTION

Being comprised largely of postmitotic neurons that must persist for a lifetime, the central nervous system is at risk for damage that would be less dire in tissues wherein stem cells can be effectively mobilized. The accumulation of damage has important implications for neurodegenerative disease pathogenesis and may be responsible for features of the aged brain below the level of overt pathology. A hallmark of aged and diseased neurons is the aggregation of damaged or misfolded proteins, and it has long been thought that loss of protein homeostasis plays an important role in neurodegenerative disease (Gray et al., 2003). Recent literature highlights the loss of RNA homeostasis as a recurrent theme in neurodegenerative disease and while it is possible to imagine how the failure of regulation at either the RNA or protein level could be catastrophic for neurons, a better course might be to consider how these failures may be related mechanistically. RNA and protein have linkages beyond the directional flow of information from DNA to RNA to protein (the “central dogma” of biology). Indeed we will argue that complexes of RNA and protein mediate all aspects of RNA metabolism (including splicing, stability, transport, and translation) and are the weak link in the maintenance of neuronal homeostasis. Crucial to these interactions are intrinsically disordered proteins. With age-related decline in proteolytic efficiency the turnover and regulation of ribonucleoprotein complexes may become increasingly dysfunctional, and what ensues may be an “error catastrophe” of a type not anticipated by Orgel when he first coined the term (Orgel, 1963). There is already substantial evidence for this model in a subset of neurodegenerative disorders, and we postulate that this may serve as a template to explain certain aspects of “non-pathological” brain aging as well.

## THE PROMINENCE OF RNA-BINDING PROTEINS IN AGE-RELATED NEURODEGENERATIVE DISEASES

Over the preceding several years RNA dysregulation has taken center stage in the pathophysiology of neurodegenerative diseases, most notably amyotrophic lateral sclerosis (ALS; Ugras and Shorter, 2012). With the exception of the roughly 20% of familial cases and less than 10% of sporadic cases of ALS attributable to mutations in the superoxide dismutase 1 (SOD1) gene (Andersen and Al-Chalabi, 2011) both sporadic and inherited forms of ALS are characterized by the aggregation of the nuclear proteins tar DNA binding protein 43 (TDP-43) or fused in sarcoma (FUS). TDP-43 and FUS are RNA-binding proteins critically involved in RNA splicing and transport. Loss of these functions is considered to be central to ALS pathogenesis. Moreover, the most common genetic basis of ALS is a hexanucleotide repeat expansion in the c9orf72 gene on chromosome 9 (Dejesus-Hernandez et al., 2011; Renton et al., 2011). The causative mutation in this form of ALS resides in an intron, resulting in the generation of an elongated RNA with pernicious properties including the ability to sequester essential RNA-binding proteins (Mori et al., 2013a). The potential also exists for protein-mediated mayhem from the intronic repeat, which can be translated through a non-ATG initiated mechanism to generate potentially toxic dipeptide repeat proteins (Mori et al., 2013b). The situation bears some similarity to that of the expanded CAG repeat in the huntingtin gene (the genetic basis of Huntington’s disease) where an RNA-mediated mechanism has only recently been revealed. Binding of the expanded CAG repeat to a translational regulatory complex containing the MID1 protein was found to enhance the translation of mRNAs containing such repeats, promoting the accumulation and aggregation of abnormal protein (Krauss et al., 2013). In both cases further work will be required to establish the relative contributions of RNA and protein-mediated mechanisms.

Other RNA-mediated diseases provide a paradigm for understanding a potential role for RNA-binding proteins in neurodegenerative pathogenesis. These disorders include myotonic dystrophy (DM; types 1 and 2), fragile X-associated tremor ataxia syndrome (FXTAS), spinocerebellar ataxia types 3, 8, 10, and 12, Huntington’s disease like 2 (reviewed in Todd and Paulson, 2010), and chromosome 9-linked frontotemporal dementia/ALS (Todd and Paulson, 2010; Renton et al., 2011). Unlike their counterparts caused by coding region trinucleotide repeat expansions, they are all caused by nucleotide repeat expansion mutations in the 5′ UTR, 3′ UTR or intronic sequences of mRNAs. The resulting expansion alters the RNA which forms complex secondary structures including hairpin loops, rendering them prone to aggregation. Indeed, the formation of microscopically visible “RNA foci” represents a histomorphological hallmark of this family of diseases (Taneja et al., 1995). These expansion-induced alterations also confer upon the mutant RNA a “gain-of-toxic function”. This RNA-induced toxicity is mediated in large part by the sequestration of RNA-binding proteins, most notably RNA splicing factors, with consequent widespread mis-splicing events. DM1, characterized by muscle wasting, myotonia, insulin resistance, cardiac conduction defects, cataracts, testicular atrophy, and cognitive dysfunction, is one of the first disorders recognized as belonging to this family. It is caused by a CTG repeat expansion mutation in the 3′ untranslated region of the *DMPK* gene (Mahadevan et al., 1992). The expanded RNA sequesters RNA splicing factors including members of the muscle-blind-like family of proteins, MBNL 1, 2, and 3 (Miller et al., 2000), disrupting their normal subcellular distribution, and usurping their function. In addition, there is pathological upregulation of another RNA-binding protein and alternative splicing factor CUG-binding protein 1 (Gallo and Spickett, 2010). These RNA splicing factors are involved in the alternative splicing of the muscle-specific chloride channel (Kino et al., 2009), insulin receptor (Paul et al., 2006), and cardiac troponin-T (Warf and Berglund, 2007) transcripts; thereby providing a unifying molecular substrate for the seemingly disparate multisystemic manifestations including myopathy, insulin resistance, cardiac conduction defects, respectively. DM1 is also characterized by mis-splicing of neuronal transcripts including those encoding the NMDAR1 glutamate receptor subunit, the microtubule associated protein tau, and the amyloid precursor protein (Jiang et al., 2004). Accordingly, DM1 is considered by many to be at least partly a neurodegenerative disorder as patients develop tau-positive neurofibrillary tangles in the brain and cognitive dysfunction (Sergeant et al., 2001). Tau-positive neurofibrillary pathology is not only encountered in disease states, but is also an accompaniment of “normal” aging. Thus, the pathogenesis of DM1 provides a salient example of how dysregulation of RNA-binding protein function can culminate in cellular changes with important implications for neurodegeneration as well as for brain aging.

The factors underlying the particular vulnerability of the central nervous system (CNS) to RNA-mediated toxicity remain to be defined. Indeed, the majority of RNA-mediated diseases affect predominantly or even exclusively the CNS in an age-dependent manner. For example, FXTAS is manifest clinically as late adult onset ataxia and cognitive decline (Jacquemont et al., 2003). It is caused by an expanded (permutation) CGG repeat (50–200; “pre-mutation”) in the 5′ untranslated region of the *FMR-1* gene (Hagerman and Hagerman, 2004). Pathologically, there is neuronal loss with widespread glial and neuronal ubiquitinated intranuclear inclusions (Greco et al., 2002). The mutant expanded RNA sequesters at least two RNA-binding proteins, hnRNA2/B1 and Pur alpha and causes their dysfunction (Jin et al., 2007; Sofola et al., 2007). Interestingly, MBNL-1 has also been described in FXTAS inclusions (Iwahashi et al., 2006). The concept of selective vulnerability of the CNS to RNA-mediated pathology can be extended even further to cell populations within the CNS. For example, returning to ALS, why motor neurons appear to be particularly susceptible to the pathogenic consequences of RNA dysregulation is an area of active investigation. Whether the demonstration that the novel RNA-binding protein RBM45, is sequestered by the neuronal inclusions in this disease has relevance for the vulnerability of motor neurons remains to be determined (Collins et al., 2012).

## RNA-BINDING PROTEINS ARE INTRINSICALLY DISORDERED PROTEINS, AND SERVE AS CRITICAL HUBS

The conventional manner of conceptualizing the operation of proteins within cells is in terms of structure-function relationships. In other words, it is generally imagined that upon translation a protein will adopt some stable structure, which will allow it to carry out its function as an enzyme or structural component. The “lock and key” metaphor is frequently invoked to describe the interaction of an enzyme with its substrate (or an antibody with its antigen), though it is recognized that the metaphor is inadequate in terms of the flexibility of proteins and their propensity to undergo structural changes in response to substrate binding and/or post-translational modifications. This textbook notion of the structure/function relationship is challenged by the growing catalog of proteins that are intrinsically disordered, in whole or in part. If the definition of an intrinsically disordered protein (IDP) restricts inclusion to those proteins containing disordered segments of 30 or more residues, roughly a third of eukaryotic proteins would qualify (Ward et al., 2004). Importantly, IDPs are over-represented among the family of aggregation-prone proteins implicated in neurodegenerative diseases. Disordered segments can be predicted with increasing accuracy by web-based algorithms such as PONDR-Fit (Xue et al., 2010), DISOPRED2 (Ward et al., 2004), and FoldIndex (Prilusky et al., 2005). These predictions are supported by abundant *in vitro* evidence from structural analytic methodologies such as circular dichroism, solution state NMR, and small angle X-ray scattering (Kriwacki et al., 1997; Dyson and Wright, 2004; Li and Song, 2007; Binolfi et al., 2012; Tamiola and Mulder, 2012). A recurrent feature of disordered segments that can be exploited in search algorithms is the distortion of amino acid frequencies in favor of hydrophilic and charged residues (at the expense of bulky hydrophobic residues) relative to the entire proteome. It is unlikely that a stably folded structure can form in the absence of a hydrophobic core, and charged side groups will work against the compaction of protein structure.

Whereas some IDPs appear to execute their function in the unstructured state (for example the nuclear pore complex proteins that form an unstructured “gel” to preclude the unassisted passage of other proteins through the pore; Alber et al., 2007; Meinema et al., 2011) others undergo a transition to a folded state upon binding of specific substrates. A well characterized example of this is the p27 (Kip1) protein, whose function is to inactivate a number of cyclin-dependent kinases (CDKs) under conditions where cell growth and division are undesirable. The p27 protein adopts a stable structure upon CDK binding, but that structure is dictated by the particular CDK that is bound (Galea et al., 2008). One CDK inhibitor is thereby able to interfere with the activity of a number of substrates of differing topology (Yoon et al., 2012). The p53 protein provides another remarkable example of substrate-induced structural pleiotropy – depending on its interaction partner the same intrinsically disordered segment of p53 can adopt the structure of an alpha helix, a beta sheet, or of various stable folds (Uversky et al., 2008).

Because IDPs have the potential to bind multiple partners with high specificity but low affinity they are thought to be ideally suited to serve as hub proteins in scale-free networks (the existence of hubs is the defining feature of a scale-free network). Hubs increase the robustness of networks; random node failure is less deleterious to highly connected networks containing hubs, a property exploited in communications networks such as the internet. Hubs also serve to shorten the distance between any two nodes, increasing the efficiency of network operations (these concepts are reviewed in a biological context in Dunker et al., 2005). Though hubs provide these benefits, they are of disproportionate importance to networks, and in a biological system the loss of a hub protein has the potential to be lethal [Jeong et al. (2001) provide genetic evidence for the criticality of hub proteins in the yeast model system]. The integration of signals and delivery of outputs may require the assembly of multi-component complexes, and hub proteins often serve as scaffolds for such complexes. The expectation would therefore be that there should be a correlation between the extent of a protein’s disorder and its participation in complex formation, a prediction borne out by computational analysis of test proteomes (Iakoucheva et al., 2002; Hegyi et al., 2007; Schlessinger et al., 2007). It is also to be expected that more elaborate signaling networks would be required for the additional demands of multicellularity and development in metazoans, and that with increased requirement for hubs the level of protein disorder should be higher in eukaryotes as a class than in prokaryotes. This prediction is also supported by proteome analysis (Schad et al., 2011). Finally, with their promiscuous binding it is reasonable to imagine that the abundance of hub proteins should tightly regulated. Thus, if IDPs are disproportionately represented in hubs their levels may be lower than the remainder of the proteome, another prediction in agreement with the available data (Gsponer et al., 2008; Vavouri et al., 2009).

Within the cell RNA molecules exist as topologically complex entities with extensive hairpins and loops dictated by intrastrand base pairing (for a review see; Holbrook, 2005). RNA is subject to many levels of metabolic regulation, influencing its splicing, stability, subcellular localization, and translational efficiency. RNA regulation is imposed through interacting proteins, but it is inconceivable that each unique RNA structure could be recognized by a set of interacting proteins through “lock and key” mechanisms (this would require many more RNA-binding proteins than there are RNA transcripts). The solution to the problem is for RNA-binding proteins to be IDPs, typically utilizing unstructured segments for RNA recognition, and folding upon binding to their RNA interaction partners (Battiste et al., 1996; Mogridge et al., 1998). Indeed, an analysis of over 200,000 proteins from the Swiss-Prot database using the PONDR VL3E predictor of long disordered segments identified the functional category ribonucleoproteins as most strongly correlated with disorder (Xie et al., 2007). These proteins contain RNA recognition motifs (RRMs; Query et al., 1989; Clery et al., 2008) and regions rich in the simple sequence arginine, glycine, glycine (denoted RGG in the single letter code; for this reason these elements are referred to as RGG motifs; Ohno et al., 1994; Rajyaguru and Parker, 2012). Examples of mammalian RGG motif proteins include hnRNP proteins (Kiledjian and Dreyfuss, 1992; Siomi and Dreyfuss, 1995) and the Sm (Brahms et al., 2001; Friesen et al., 2001) proteins involved in mRNA splicing. In the context of neurodegenerative disease, the prominent examples are the TDP-43 (Dammer et al., 2012) and FUS/TLS (Sun et al., 2011) proteins, implicated in ALS and in frontotemporal dementia in a mutually exclusive fashion.

**FIGURE 1:**
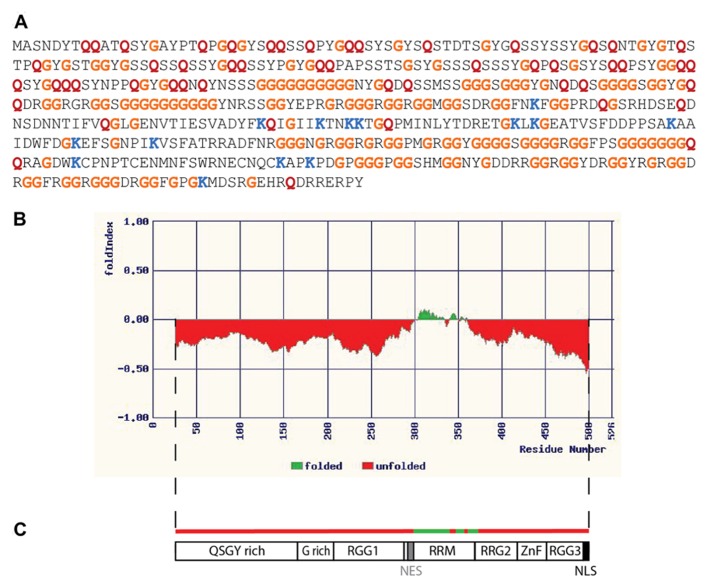
**Fused in sarcoma as an intrinsically disordered RNA-binding protein.** **(A)** Primary sequence of the 526 amino acid isoform of human FUS. Relative to all predicted protein sequences (using data from the UniProt KB/Swiss-Prot database, release 2011_09) the FUS sequence contains an unusually high proportion of some residues, while being deficient in others. Some examples are indicated by colored letters: glycine (G, orange) and glutamine (Q, red) are more abundant than expected, representing 28.7% of FUS resides as opposed to 7.1% in database proteins in the case of glycine, and 9.9% of FUS resides as opposed to 3.9% in database proteins in the case of glutamine. Lysine, on the other hand, is less abundant than the average, representing only 2.7% of FUS residues as opposed to 7.2% of proteins in the database. **(B)** Disorder in the FUS protein as predicted by the FoldIndex algorithm (available at ). Only a central portion of approximately 50 residues (indicated in green) is predicted to have a stably folded structure. **(C)** Domain organization of FUS. The folded domain corresponds to a region with nucleic acid binding properties (designated the RRM, or RNA recognition motif, though this region may also bind DNA). There are regions rich in the amino acids indicated by their single letter codes, as well as domains rich in the simple repeat arginine, glycine, glycine (RGG). ZnF indicates a putative zinc finger motif (Iko et al., 2004). NES represents a region with nuclear export activity, while NLS represents the nuclear localization signal. This schematic is adapted from a similar figure in the review of Lagier-Tourenne and Cleveland (2009).

It is beyond the scope of the current discussion to elaborate upon all of the ways in which disordered RNA-binding proteins can serve as critical network nodes, but as an illustrative example consider the case of FUS/TLS (hereafter simply FUS). FUS is a conserved protein whose orthologs have been identified in the fruit fly (designated cabeza, or caz; Stolow and Haynes, 1995) and nematode FUST-1 (accession number NP_495483). The largest human isoform of FUS is a ubiquitously expressed 526 amino acid protein whose primary sequence is obviously unusual (**Figure 1A**). The protein is predicted to be almost entirely disordered by the available algorithms (**Figure 1B**), with the exception of a small central domain. The central segment of FUS is thought to mediate binding to nucleic acids (**Figure 1C**); one of the known functions of FUS is as a DNA binding transcription factor (Uranishi et al., 2001; Tan et al., 2012). The organization of FUS with a structured central domain and flanking unstructured regions resembles that of p53 (Uversky and Dunker, 2010), whose repertoire of functions includes transcription factor activity (Beckerman and Prives, 2010). The preponderance of FUS publications, however, relate to its various roles in RNA metabolism. FUS is a nuclear/cytoplasmic shuttling protein (Dormann et al., 2010), with established roles in both subcellular compartments. In the nucleus (where the bulk of FUS protein normally resides; Dormann et al., 2010) FUS associates with the spliceosomal complex (Meissner et al., 2003) and is required for the correct processing of mRNAs such as that encoding Tau (Orozco et al., 2012). In the brain FUS associates with nascent RNA transcripts and regulates alternative splicing (Ishigaki et al., 2012; Rogelj et al., 2012). Depletion of FUS by stereotactic delivery of antisense oligonucleotides into the adult mouse brain has been shown to alter the splicing of nearly 1,000 transcripts (Lagier-Tourenne et al., 2012). Nuclear FUS also plays a role in telomere maintenance by binding to G-quadruplexes (stacked tetrads of guanine residues) that exist in telomeric DNA and in telomeric repeat-containing RNAs associated with the telomerase complex (Takahama et al., 2013). The formation of G-quadruplexes has recently been documented in the disease-associated G-rich repeats of the C9orf72 RNA, which have been shown to associate with a subset of RNA-binding proteins (Reddy et al., 2013). It is not yet known whether FUS is a member of this group or to what extent the quadruplex figures in C9orf72-mediated pathology. In the cytoplasm of neurons FUS is involved in RNA transport to synapses (Fujii and Takumi, 2005) and translational regulation at this site. Mice lacking FUS have abnormal dendritic morphology and a decrease in the number of dendritic spines (Fujii et al., 2005). FUS does not bind all RNAs, but binds a specific subset, many of which contain a GUGG motif (Lerga et al., 2001; Lagier-Tourenne et al., 2012). Many of the RNAs bound by FUS encode products that are themselves associated with transcriptional or post-transcriptional regulation (Colombrita et al., 2012; Lagier-Tourenne et al., 2012). Overexpression of FUS is toxic to cells in culture and to neurons *in situ *(Mitchell et al., 2012), promoting excessive accumulation of FUS in the cytoplasm and the formation of cytoplasmic aggregates of characteristic appearance. The toxicity of overexpressed FUS has similarities to the toxicity of the mutant isoforms of FUS identified in human ALS; pathogenic FUS mutations typically affect the C terminal region of FUS (Mackenzie et al., 2010), which is required for nuclear localization (Dormann et al., 2010). There is evidence that RNA-binding is critical for FUS toxicity: mutations affecting RNA binding eliminate FUS aggregation and toxicity in a yeast model system (Sun et al., 2011) and toxicity can be suppressed by co-expression of RNA-binding components of mRNPs and stress granules (Ju et al., 2011; Sun et al., 2011). In summary then, FUS is an IDP that influences RNA homeostasis both in the nucleus and cytoplasm through its interactions with RNA and a large number of RNA-binding partners. FUS has all the expected properties of a hub as outlined above: it is largely disordered and binds multiple partners with high specificity, its abundance is limited, and it receives input from multiple signaling networks. As a hub protein FUS is indeed a critical node; dysregulation of FUS adversely affects RNA metabolism at multiple levels and results in neurodegenerative disease.

## IDPs ARE PROTEASOME SUBSTRATES, AND ARE VULNERABLE TO AGE-RELATED PROTEOLYTIC DEFICIENCY

Eukaryotic cells possess two major proteolytic systems, both of which are essential for neuronal homeostasis (Rubinsztein, 2006). The autophagic system utilizes membrane-delimited vesicles to recycle cell constituents into their component parts (Harris and Rubinsztein, 2012), but does so relatively slowly (it may take hours to engulf and disassemble a complex structure such as a mitochondrion). Because its substrates must be enclosed in membranes autophagic degradation is restricted to cytoplasmic constituents. The ubiquitin/proteasome system (UPS) operates in the nucleus and cytoplasm, and is responsible for the rapid degradation of individual proteins rather than more complex assemblages (Schwartz and Ciechanover, 2009). The UPS can display a high degree of specificity, which is attributable to the large number of enzymes dedicated to substrate recognition (including hundreds of ubiquitin ligases acting in concert with a much smaller number of ubiquitin conjugating enzymes to build ubiquitin chains on substrates). This elaborate enzymology orchestrates the delivery of specific substrates to the 26S proteasome, a complex molecular machine capable of unfolding incoming substrates and cleaving them into short peptides. Ubiquitin chain recognition and adenosine triphosphate (ATP)-dependent unfolding activity reside within the 19S lid structures of the 26S proteasome (Gallastegui and Groll, 2010). The active proteases reside within the interior of the 20S core proteasome, a barrel-shaped structure with narrow portals at each end. These entry pores can be gated by the disordered tails of subunit proteins (Groll et al., 2000). Folded proteins cannot pass through these pores, which can only accommodate a linear polypeptide chain. The cell contains an appreciable number of 20S core proteasomes, whose only substrates can be disordered proteins, or disordered segments of proteins. Such substrates could be degraded without prior ubiquitination (an energy-dependent activity that directs substrates to the 26S, not the 20S proteasome) and without unfolding; degradation of IDPs would therefore occur without any cost in ATP hydrolysis. It is therefore very straightforward to assay for this form of degradation *in vitro* – it will occur when substrates are mixed with purified 20S proteasomes. Testing of specific substrates predicted to be IDPs (the p21 protein, or α-synuclein, for example) has confirmed that they are efficiently degraded in this fashion (Sheaff et al., 2000; Tofaris et al., 2001; Baugh et al., 2009). Susceptibility to degradation by the 20S proteasome has been proposed as the *operational* definition of an IDP (Tsvetkov et al., 2008), an experimental approach to complement physical and computational methodologies. Thermal stability is a second easily measured parameter which correlates well with susceptibility to 20S proteasomal degradation *in vitro* (Tsvetkov et al., 2012).

The vulnerability of IDPs to proteasomal degradation is such that in uncomplexed form they may be rapidly eliminated within cells; association with molecular “nannies” has been hypothesized as a protective mechanism (Tsvetkov et al., 2009). As mentioned previously the abundance of IDPs is typically low (a desirable state for proteins acting as hubs in regulatory networks), and 20S proteasomal degradation may be one mechanism that limits the abundance of uncomplexed IDPs. If so, any decline in the abundance or enzymatic efficiency of 20S proteasomes would lead to accumulation of uncomplexed IDPs, with two potentially deleterious consequences. First, one would expect perturbations in the stoichiometry of complex formation. For a single RNA-binding protein such as FUS the consequences of decreased proteasomal degradation might include alterations in transcriptional elongation, mRNA splicing, mRNA stability, mRNA export, mRNA transport, and mRNA translation! Though little is currently known about the regulation of the FUS RNA itself it is known to be a binding partner of the TDP-43 protein (Sephton et al., 2011), and it is conceivable that abnormalities in one RNA-binding protein could affect the other with more global consequences. Indeed it is easy to imagine how perturbation of FUS levels could generate a vicious cycle of RNA dysregulation. Inefficient turnover of FUS by the proteasome may also promote the formation of FUS aggregates. Molecular crowding of a protein like FUS may be sufficient to promote its aggregation (though aggregation is also influenced by post-translational modification of FUS including arginine methylation; Dormann et al., 2012). Once formed, aggregates of RNA-binding IDPs may be difficult for the proteasome to disassemble and degrade; autophagy may be the only option for clearance of aggregated IDPs (**Figure 3B**). There is evidence that pharmacological enhancement of autophagy reduces the number of inclusions and corresponding loss of motor function in a transgenic model of TDP-43 proteinopathy (Wang et al., 2012).

**FIGURE 2:**
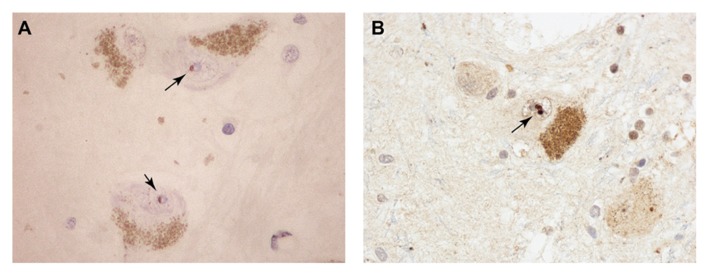
**Pigmented dopaminergic neurons in the substantia nigra from aged, non-diseases subjects containing intranuclear MBs (arrows) immunoreactive for promyelocytic leukemia protein (PML; A) and ubiquitin (B)**.

The event that could trigger these proposed molecular cataclysms may be nothing more than an age-related decline in proteasomal activity. Indeed the conditional knockout of a proteasome subunit in motor neurons effectively phenocopies ALS in mice, with aggregation of TDP-43 and FUS positive inclusions in spinal motor neurons (Tashiro et al., 2012). Knockout of an autophagic component did not have this effect. The age-related decline of ubiquitin-mediated proteolysis has been well documented in the mammalian brain (reviewed in Gray et al., 2003). Even in the “healthy” aging brain (in individuals not diagnosed with neurodegenerative disease) there are indications of pathologic change. Of particular relevance to the ubiquitin-proteasome system is the age-related accumulation of ubiquitin-immunoreactive neuronal inclusion bodies which share features with their counterparts in neurodegenerative disorders (Dickson et al., 1990; Gray et al., 2003). Marinesco bodies (MBs) provide a morphological metaphor for the blurred interface between normal and pathological brain aging. MBs are spherical intranuclear inclusions (**Figure 2**) found in the catecholaminergic neurons of the substantia nigra and locus coeruleus of the non-diseased primate brain (Yuen and Baxter, 1963). Their frequency increases significantly with age. In addition to ubiquitin, these structures contain a variety of additional UPS-related proteins including p62, EDD1, NEDD8, NUB1, SUMO-1, and SUMO-2 (Odagiri et al., 2012). Despite their morphological and biochemical similarity to the intranuclear inclusion bodies that characterize some neurodegenerative disorders, including the polyglutamine repeat disorders and neuronal intranuclear inclusion body disease, they have long been considered inert, non-pathological entities. More recent studies, however, have demonstrated that their appearance correlates with age-associated dopaminergic denervation of the striatum (Beach et al., 2004) and nigral neuronal degeneration (Kanaan et al., 2007). Thus, MBs may either directly impose, or alternatively represent markers of, pernicious cellular events related to proteolytic failure in the aging CNS. Whether the same holds true for the numerous other types of ubiquitin-positive inclusions that have been described in the aging non-diseased human brain (Gray et al., 2003) is unknown. Moreover, whether MBs sequester RNA-binding proteins has not been studied. However, studies have demonstrated a significant increase in their frequency in the RNA-mediated disorder DM (Ono et al., 1987). The apparent stochasticity of this process may reflect the fact that in the otherwise healthy aging brain individual neurons may reach the “tipping point” for the RNA crisis at different times.

**FIGURE 3:**
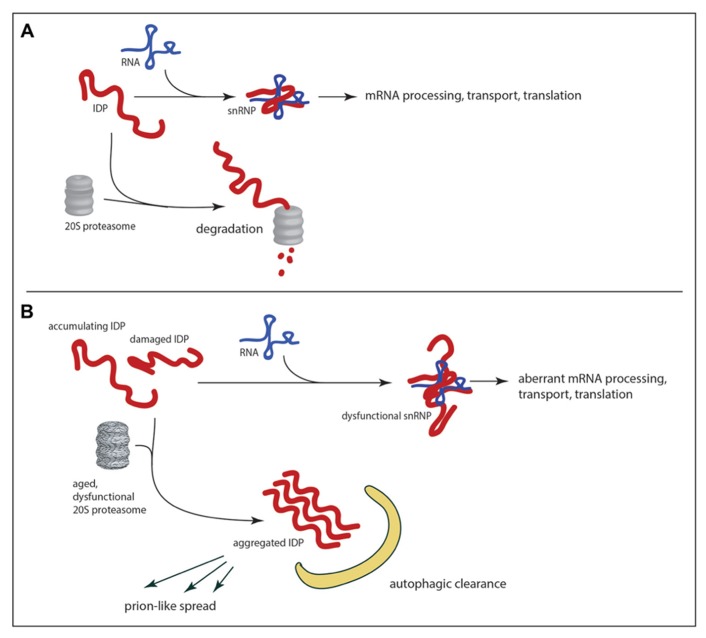
**RNA-binding IDPs as a weak in link in age-related neurodegeneration.** In healthy neurons **(A)** the abundance of IDPs is limited by the 20S proteasome, which can mediate proteolytic degradation in a ubiquitin-independent manner. With the decline of proteolytic efficiency in the aged brain **(B)** there is accumulation of RNA-binding IDPs, which can promote aggregation and/or abnormalities in RNA metabolism. The potential exists for feedback loops of escalating dysfunction as abnormal IDPs are produced. If aggregates are not cleared by autophagy the potential also exists for the spread of dysfunction to other neurons through prion-like mechanisms.

## PERTURBATION OF RNA HOMEOSTASIS MAY SPREAD THROUGH PRION-LIKE MECHANISMS

Age-related decline in protein degradation may contribute to an RNA homeostatic crisis by promoting the accumulation of RNA-binding IDPs as described above, but recent speculation on mechanisms by which the pathology of neurodegenerative diseases may spread within an individual has increased the potential for IDP-mediated devastation. It has long been known that pathological changes in the nervous system follow anatomical patterns of spread that are stereotypic for each disease. In the case of sporadic Parkinson’s disease, for example, Braak et al. (2002) have proposed a staging system wherein alpha-synuclein aggregates in the olfactory neurons and brainstem are detected at an early stage. Only at later stages is there detection of Lewy bodies in the substantia nigra (the hallmark of PD). Braak has proposed that the initial insult may occur at distant sites in the gut or olfactory epithelium, and may spread via long unmyelinated axons to the brain (Hawkes et al., 2007). Although this mechanism is still very controversial, similar mechanisms have been proposed to explain temporal changes in cortex and striatum that are characteristic of Huntington’s disease and the spread of neurofibrillary tangles from hippocampus and associated structures to the neocortex in Alzheimer’s disease (Brundin et al., 2010; Cushman et al., 2010; Guest et al., 2011; Walker and Levine, 2012). The hypothesized mechanism of spread in all cases is cell to cell transmission of a prion-like entity: a small aggregate that would be released by exocytosis on exosomes, cell lysis or transmitted by other means (for example direct transfer through nanotubes; Gousset et al., 2009). There is accumulating evidence that protein aggregates can be taken up by cells (Lee et al., 2008; Angot et al., 2012), and once this occurs it is plausible that the incoming aggregate would seed further aggregation in the recipient cell. Could the intrinsically disordered, aggregation-prone RNA-binding proteins be transmitted in this fashion? Already there is both computational and experimental evidence in favor of this idea. A hidden Markov model algorithm has been developed based on known yeast prion sequences (Alberti et al., 2009). When applied to the human proteome this algorithm ranked FUS protein 15th in its list of predicted prions (Cushman et al., 2010). The TDP-43 protein has been shown to template prion-like self-assembly *in vitro *(Furukawa et al., 2011). It remains to be demonstrated that RNA dysregulation can be transmitted from cell to cell through aggregated RNA-binding IDPs, but this would provide a route through which a rare stochastic calamity in one cell could be propagated to adjacent (and perhaps distant) bystanders (**Figure 3**). It may be that age-related decline in proteolytic capacity could trigger the initial aggregation of the IDP, even in the absence of somatic mutations.

## CONCLUSION

Based on the current literature we suggest that RNA homeostasis is a weak link in the aging brain, and the loss of RNA homeostasis underlies much neurodegenerative pathology. The precipitating event for an ensuing catastrophe may be the well-documented decline in proteolytic efficiency which would have immediate and deleterious effects on substrates such as the RNA-binding IDPs. In such a scenario the initiation of an “error catastrophe” in which aggregation of RNA-binding proteins promotes dysregulation of RNA splicing, RNA stability, RNA export, transport and translation would have knock-on effects on protein structure and function. The added burden to the proteasome and compromised function of proteolytic components would result in further perturbation of RNA homeostasis in a self-amplifying cycle. This catastrophe may be exported to neighboring cells through the prion-like spread of ribonucleoprotein complexes. We believe that the hypothesized mechanism of age-related neurodegeneration is experimentally tractable, and critical aspects of the hypothesis can be tested using mouse models and cell culture systems.

## Conflict of Interest Statement

The authors declare that the research was conducted in the absence of any commercial or financial relationships that could be construed as a potential conflict of interest.

## Abbreviations

ALS: amyotrophic lateral sclerosis
ATP: adenosine triphosphate
CDK: cyclin-dependent kinase
CNS: central nervous system
DM: myotonic dystrophy
FXTAS: fragile X-associated tremor ataxia syndrome
IDP: intrinsically disordered protein
MB: Marinesco body
RNP: ribonucleoprotein
RRM: RNA recognition motif
UPS: ubiquitin/proteasome system
UTR: unstranslated region
